# Method for Estimating Three-Dimensional Knee Rotations Using Two Inertial Measurement Units: Validation with a Coordinate Measurement Machine

**DOI:** 10.3390/s17091970

**Published:** 2017-08-27

**Authors:** Rachel V. Vitali, Stephen M. Cain, Ryan S. McGinnis, Antonia M. Zaferiou, Lauro V. Ojeda, Steven P. Davidson, Noel C. Perkins

**Affiliations:** 1 Department of Mechanical Engineering, University of Michigan, Ann Arbor, MI 48109, USA; smcain@umich.edu (S.M.C.); lojeda@umich.edu (L.V.O.); stevepd@umich.edu (S.P.D.); ncp@umich.edu (N.C.P.); 2 Department of Electrical and Biomedical Engineering, University of Vermont, Burlington, VT 05405, USA; rsmcginn@uvm.edu; 3 Department of Orthopedic Surgery, Rush University Medical Center, Chicago, IL 60612, USA; antonia_zaferiou@rush.edu

**Keywords:** knee rotations, inertial measurement units, wearable sensors, human performance, motion tracking

## Abstract

Three-dimensional rotations across the human knee serve as important markers of knee health and performance in multiple contexts including human mobility, worker safety and health, athletic performance, and warfighter performance. While knee rotations can be estimated using optical motion capture, that method is largely limited to the laboratory and small capture volumes. These limitations may be overcome by deploying wearable inertial measurement units (IMUs). The objective of this study is to present a new IMU-based method for estimating 3D knee rotations and to benchmark the accuracy of the results using an instrumented mechanical linkage. The method employs data from shank- and thigh-mounted IMUs and a vector constraint for the medial-lateral axis of the knee during periods when the knee joint functions predominantly as a hinge. The method is carefully validated using data from high precision optical encoders in a mechanism that replicates 3D knee rotations spanning (1) pure flexion/extension, (2) pure internal/external rotation, (3) pure abduction/adduction, and (4) combinations of all three rotations. Regardless of the movement type, the IMU-derived estimates of 3D knee rotations replicate the truth data with high confidence (RMS error < 4° and correlation coefficient r≥0.94).

## 1. Introduction

The human knee is susceptible to injury from multiple mechanisms including, for example, hyperextension (including varus and valgus components), over-use, and direct impact (see, for example, [1,2]). Knee injuries are also known to induce osteoarthritis and may severely limit activities of daily living [3,4]. Accordingly, the three-dimensional rotations across the knee (flexion/extension, internal/external rotation, and abduction/adduction) serve as important markers of knee health and performance and in multiple contexts such as human mobility, worker safety and health, athletic performance, and warfighter performance. For example, clinicians observe knee rotations to diagnose knee injuries and to assess the need for surgical interventions (such as knee arthroplasty), recovery, and physical therapy [5,6]. Many biomechanical analyses incorporate measurements of knee rotations including studies of the long-term effects from knee injuries [7,8], joint disorders including arthritis [9] and age- and gender-related differences in knee health [10]. By analyzing knee rotations, researchers have also explored the effects of load carriage related to fall prevention [11], metabolic cost during walking [12,13], and warfighter performance during walking [14]. Warfighter performance is particularly difficult to study since conditions under which warfighters perform cannot be recreated well in a laboratory. For example, unconstrained walking gait over ground differs from gait employed while walking on a treadmill [15].

Two commonly used methods for measuring knee rotations, namely goniometers and optical motion capture, are largely restricted to laboratory settings. Both uniaxial goniometers for measuring flexion/extension [5,9] and triaxial goniometers for measuring flexion/extension, internal/external rotation, and abduction/adduction [16] mount externally across the knee. In addition to errors induced by soft tissue sensor mounting, errors may also arise from misalignment of the goniometer sense axes relative to the knee anatomical axes. Knee rotations are also frequently deduced from optical motion capture data using markers attached directly to soft tissue (see, for example, [6,8,11,14,17]). Motion capture methods place significant limits on the capture volume and may also suffer from occasional marker occlusion, soft tissue movement relative to the underlying skeletal structure, and marker placement precision on approximate bony landmarks.

Body-worn inertial measurement units (IMUs) provide an alternative means to estimate the three-dimensional rotations across the knee from data collected from the on-board accelerometers, angular rate gyros, and magnetometers (if available). Unlike the above methods that are largely restricted to laboratory settings, body-worn IMUs may readily be used outside the laboratory, thereby potentially increasing the validity of research conclusions by enabling data collections in the real world. To estimate 3D rotations across the knee, one must first estimate the relative orientation of the anatomical axes of the thigh to those of the shank, a result that does not immediately follow from shank- and thigh-mounted IMUs as they are independent devices. However, this result is obtainable if the orientation of each IMU is first established relative to a (common) world frame of reference, which is a technical challenge addressed in this paper.

A number of prior studies propose strategies for estimating rotations across joints using body-worn IMUs. Focusing on the human wrist, Luinge et al. [18] deduce the relative orientation of two IMUs by exploiting a wrist joint constraint. Similarly, Müller et al. [19] determine the rotation axes for and ultimately the angles across the elbow via an optimization algorithm built on the condition that it is a two degree-of-freedom joint. Using the medial/lateral axis of the knee as an anatomical constraint, Cooper et al. [20] estimate (planar) knee flexion/extension during straight-line walking through fast running. Following suit, Seel et al. [21] also estimate knee flexion/extension after first constructing the knee medial/lateral axis using angular rate data from shank- and thigh-mounted IMUs assuming their world frames are identical. In addition, they fuse gyro- and accelerometer-based knee flexion/extension estimates to significantly reduce the effects of drift during long trials. An extension is offered by Laidig et al. [22] where knee flexion/extension angles are accurately estimated by exploiting the knee’s hinge axis to control misalignment about the vertical axis due to drift and/or magnetic field interference. By contrast, Favre et al. [23] exploit the anterior/posterior axis of the knee as an anatomical constraint to also estimate knee internal/external rotation and abduction/adduction (i.e., full 3D rotations across the knee) through an intermediate step employing the distinct world frames of the two IMUs. The misalignment between the distinct world frames is estimated using an assumed constant correction angle for relatively short duration trials. An extension is offered by Brennan et al. [24] through a time-varying correction angle using correction estimates at the start and the end of each trial and by requiring the IMUs return to their original orientations. This method is validated using an instrumented gimbal that provides ground truth data from embedded optical encoders. While the above methods do not consider IMUs that include magnetometers, other methods do so and use magnetic north to align the IMU world frames as well to estimate (yaw) drift about the vertical axis [25,26]. An overall approach to fusing magnetometer and inertial sensor data is outlined in [27] which also considers corrections for magnetic field interference. However, the estimates of magnetic north from two IMUs may differ due to discrepancies in magnetometer data [28,29] despite these corrections, ultimately limiting this advantage. Reducing these discrepancies may follow from improving IMU hardware, updating filter parameters, or including additional (and complementary) sensors for fusion [30].

This study serves as an extension to those above by presenting, and carefully validating, a new method to estimate 3D rotations across the knee using a pair of shank- and thigh-mounted IMUs. Although this study employs IMUs with magnetometers, the method herein applies equally well to IMUs without magnetometers. Like [20,21,22], we use the medial/lateral axis of the knee as a kinematic constraint. However, in addition to estimating knee flexion/extension, our method also successfully estimates internal/external rotation and abduction/adduction. In so doing, the distinct world frames of the two IMUs are aligned by exploiting a vector form of the constraint equation in [21] that the medial/lateral axis have identical orientation in the two world frames. This constraint yields the (dynamic) correction needed to align the two world frames during, and also between, time intervals when the knee is functioning predominantly as a hinge joint, but without any requirement that the knee return to its initial orientation as in [24]. We open this paper with an overview of this new method together with a summary of companion experiments conducted on a coordinate measuring machine that yields high-precision truth data for validation.

## 2. Materials and Methods

**Figure 1:**
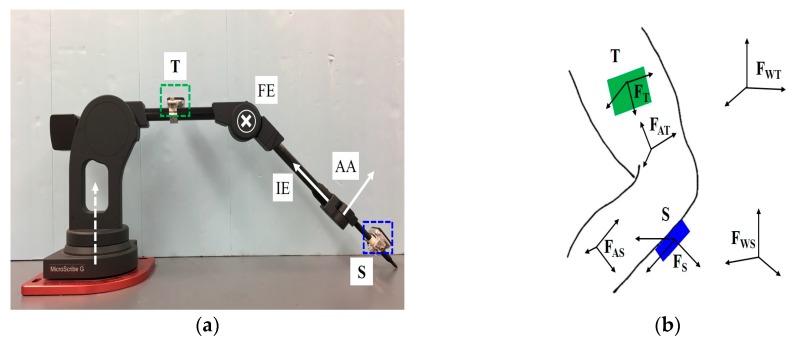
Knee analog formed by a coordinate measuring machine (CMM). (**a**) Three anatomical axes for flexion/extension (FE), internal/external rotation (IE), and abduction/adduction (AA) are labeled at the corresponding rotational joints of the CMM. Two labeled IMUs are mounted to the CMM with T (green) analogous to a thigh-mounted IMU and S (blue) analogous to a shank-mounted IMU. (**b**) Definitions of three frames of reference for a human knee associated with a shank-mounted IMU (blue) including the shank IMU frame, $F_{S}$, the shank anatomical frame, $F_{AS}$, and the shank IMU’s world frame, $F_{WS}$. Analogous frames of reference are illustrated for the thigh-mounted IMU (green).

As stated above, the objective of this paper is to present a new method for estimating 3D rotations across the knee that employs a kinematic constraint imposed by the knee’s structure to remove drift and to carefully benchmark the accuracy of these estimates. To this end, we employ a coordinate measurement machine (CMM) (MicroScribe G2X, Solution Technologies, Oella, MD, USA) that functions as a knee analog and that provides truth data for validation. The CMM shown in Figure 1a embeds high precision optical encoders (0.0003° resolution [31]) that measure rotations about three axes representing knee flexion/extension (FE), internal/external rotation (IE), and abduction/adduction (AA). Two IMUs (Opal sensors, APDM, Portland, OR, USA; Sensor characteristics and orientation estimate information available at http://www.apdm.com/wearable-sensors/), rigidly mounted to the illustrated two links, replicate the functions of thigh- and shank-mounted IMUs (T and S, respectively). The resulting apparatus enables direct comparison of IMU-estimated knee flexion/extension, internal/external rotation, and abduction/adduction to measured values from the three high precision optical encoders and over a wide range of simulated 3D knee movements.

### 2.1. Experimental Procedure

Data from the two IMUs are first time-synchronized to the encoder data from the CMM. The assembly is rotated by hand about the CMM’s base (white axis in Figure 1a) with the three knee axes (FE, IE, AA) locked. The angle measured by the optical encoder about the base (dashed white) axis is differentiated with respect to time yielding an angular velocity signal to compare with those measured by the thigh (green) and shank (blue) IMUs. The data from the two IMUs are already time-synchronized, and their synchronization with the data from the CMM follows from measuring (and subsequently subtracting) the time delay between their respective angular rates.

Next, we complete two functional alignment movements that pre-identify the anatomical axes of the shank and thigh for the knee analog. First, the superior/inferior axes of the shank and thigh are estimated by holding each segment still (for approximately 10 s) while vertical. The measured acceleration for each segment defines the direction of gravity, which is also aligned with the superior/inferior axis of each segment. Next, the medial-lateral axis is established using essentially the same procedure outlined in [21]. In particular, the CMM is exercised purely about the flexion/extension axis with the two remaining knee axes locked. In so doing, the knee acts as a pure hinge, and one can readily compute the medial/lateral (hinge) axis with respect to the sense axes of each IMU. The resulting medial/lateral axes, so measured by the thigh- and shank-mounted IMUs, play a key role in the estimation process described below.

Finally, we consider four characteristically distinct knee movements for generating the truth data for validation. These movements, each repeated for N = 50 trials, include (1) pure flexion/extension, (2) pure internal/external rotation, (3) pure abduction/adduction, and (4) combinations of all three rotations. Each type of movement is made by hand, and one trial lasts approximately 10 s (with the appropriate CMM axes either free or locked).

### 2.2. Defining Segment Frames of Reference

Figure 1b illustrates three distinct frames of reference associated with each of the thigh- and shank-mounted IMUs. In particular, we call attention to the shank IMU frame, $F_{S}$ (defined by the IMU sense axes), the shank anatomical frame, $F_{AS}$, and the shank IMU world frame, $F_{WS}$. The three analogous frames of reference ($F_{T}$, $F_{AT},\;F_{WT}$) associated with the thigh-mounted IMU are also illustrated. The ultimate goal is to estimate the 3D rotations (FE, IE, AA) across the knee and doing so requires estimating the orientation of the shank anatomical frame, $F_{AS}$, relative to the thigh anatomical frame, $F_{AT}$. That critical step, however, requires introducing a *common* world frame of reference for the two IMUs as described below. Prior to that, we first establish the separate world frames and anatomical frames for both segments described below.

The quaternion output (which is provided by proprietary software from APDM; for examples on how to compute quaternion output from IMU data, refer to [18,20,24,25]) from an IMU say S is used to construct a direction cosine matrix (DCM) between an IMU frame, $F_{S}$, and a world frame, $F_{WS}$. The world frame $F_{WS}$ is defined by three mutually orthogonal axes (${\hat{X}}_{WS}$,$\;{\hat{Y}}_{WS},\;{\hat{Z}}_{WS}$) with the ${\hat{Z}}_{WS}$ axis chosen to align with gravity (using accelerometer data from S), the ${\hat{X}}_{WS}$-axis chosen to align with magnetic north (using magnetometer data from S), and the ${\hat{Y}}_{WS}$ axis computed from ${\hat{Y}}_{WS}={\hat{Z}}_{WS}\times {\hat{X}}_{WS}$ and thus chosen to point west. Let $R_{WS/S}$ represent the resulting DCM from$\;F_{S}$ to $F_{WS}$, a result that necessarily utilizes the magnetic north estimate from S. An analogous procedure holds for the thigh-mounted IMU leading to the construction of $R_{WT/T}$ representing the DCM from $F_{T}$ to $F_{WT}$, a result that necessarily utilizes the magnetic north estimate from T. Note also that the location and orientation of either IMU on its respective segment are arbitrary and the orientation of each IMU relative to its respective anatomical axes is established using the aforementioned functional alignment movements as detailed next.

The two functional alignment movements establish the shank anatomical axes (${\hat{X}}_{AS},\;{\hat{Y}}_{AS},{\hat{Z}}_{AS}$) that define $F_{AS}$ and the thigh anatomical axes (${\hat{X}}_{AT},\;{\hat{Y}}_{AT},{\hat{Z}}_{AT}$) that define $F_{AT}$. The procedure for both body segments is identical, and so we detail only that for the shank. First, the average acceleration measured during the still period yields a first estimate of the shank-fixed ${\hat{Z}}_{AS}$ axis (superior-inferior axis) of the shank anatomical frame that is approximately aligned with gravity. (Note that during a trial with a human subject standing still, the direction of gravity remains approximately aligned with the superior-inferior axis.) This still period is followed by rotations purely about the CMM flexion/extension axis (while locking the other two rotational degrees of freedom). Following Seel et al. [21], the resulting angular velocity ${\vec{\omega }}_{S}$ and ${\vec{\omega }}_{T}\;$measured by S and T, respectively, is used to define a unit vector ${\hat{n}}_{S}$ aligned with the medial-lateral (hinge) axis measured in $F_{S}$. The medially pointing direction of this unit vector defines the anatomical ${\hat{X}}_{AS}$ axis. The anterior-posterior axis, ${\hat{Y}}_{AS},$ follows immediately from

(1) $${\hat{Y}}_{AS}={\hat{Z}}_{AS}\;\times \;{\hat{X}}_{AS}$$

Finally, we adjust (if needed) the superior-inferior axis so that the medial-lateral axis remains orthogonal to the other two anatomical axes per,

(2) $${\hat{Z}}_{AS}={\hat{X}}_{AS}\;\times \;{\hat{Y}}_{AS}$$

The resulting orthonormal triad (${\hat{X}}_{AS},\;{\hat{Y}}_{AS},{\hat{Z}}_{AS}$), which are measured with respect to the shank IMU frame $F_{S}$, define the shank anatomical frame with the hinge axis ${\hat{n}}_{S}={\hat{X}}_{AS}$ The (constant) DCM from the shank IMU frame, $F_{S}$, to the shank anatomical frame, $F_{AS}$, follows from,

(3) RASS=[X^ASY^ASZ^AS]

where each row contains the components of the anatomical axes measured with respect to $F_{S}$. Per ISB convention [32], the medial-lateral axis corresponds to the FE axis, the anterior-posterior axis corresponds to the AA axis, and the superior-inferior axis corresponds to the IE axis. An analogous procedure establishes the knee hinge axis ${\hat{n}}_{T}=\;{\hat{X}}_{AT}$ and the thigh anatomical frame (${\hat{X}}_{AT},\;{\hat{Y}}_{AT},{\hat{Z}}_{AT}$), which are measured with respect to the thigh IMU frame $F_{T}$. The (constant) DCM from the thigh IMU frame, $F_{T}$, to the thigh anatomical frame, $F_{AT}$, follows from,

(4) RATT=[X^ATY^ATZ^AT]

### 2.3. Estimating 3D Knee Rotations Following Construction of a Common World Frame

The dynamic 3D knee rotations are ultimately estimated from the (time-varying) DCM $R{\left(t\right)}_{AT/AS}$ from the shank anatomical frame, $F_{AS}$, to the thigh anatomical frame, $F_{AT}$. One may believe that this DCM follows from the component rotations defined above per,

(5) R(t)ATAS= RATT R(t)TWTR(t)WSSRSAS

However, this result is correct only in the rare instances when the two IMU world frames,$\;F_{WS}$ and $F_{WT}$, are aligned. Frequently, the magnetometers in the two IMUs, S and T, provide distinct estimates of magnetic north (especially indoors where ferromagnetic interferences are often prevalent), and thus their respective world frames $F_{WS}$ and $F_{WT}$ will be misaligned in general. This is also true for methodologies that do not employ magnetometers, where changes in orientation are estimated from world frames constructed by other means/assumptions. Furthermore, the estimated world frames often vary with time due to sensor drift (bias) errors. In short, the two IMUs are independent sensors yielding independent and time-varying (drifting) world frames as also illustrated in the example results that follow.

Therefore, the key challenge lies in constructing a common world frame of reference for the two IMUs, or equivalently, estimating the “correction” DCM $C{\left(t\right)}_{WT/WS}$ from $F_{WS}$ to $F_{WT}$. There is no clear way of determining the angular differences in the estimates of magnetic north from the two IMUs. However, one can exploit the constraint that the hinge (medial-lateral) axes, ${\hat{n}}_{T}$ and$\;{\hat{n}}_{S}$, should be identical in a common world frame during the time intervals when the knee acts predominantly as a hinge joint. We treat this “hinge constraint” as a full vector equation and therefore generalize the scalar treatment of this constraint employed in [21]. Whenever this hinge criterion is satisfied, the correction DCM $C{\left(t\right)}_{WT/WS}$ can be constructed from ${\hat{n}}_{T}$ and ${\hat{n}}_{S}$ by computing the axis of rotation, $\hat{k},$ and the associated angle of rotation, θ, needed to align ${\hat{n}}_{T}$ and ${\hat{n}}_{S}$ in their respective world frames. To this end, the cross product,

(6) k^(t)=R(t)WSSn^S ×R(t)WTTn^T

defines the axis of rotation $\hat{k}\left(t\right)$, and the dot product,

(7) θ(t)= cos−1(R(t)WSSn^S · R(t)WTTn^T)

defines the required angle of rotation θ(t). The requisite correction DCM follows from Rodrigues’ rotation formula [33] per,

(8) $$C{\left(t\right)}_{WT/WS}=I+\sin\left(\theta \right)\tilde{k}+\left(1-\cos\theta \right){\tilde{k}}^{2}$$

where $\tilde{k}\left(t\right)$ is the skew symmetric form of $\hat{k}\left(t\right).$ The correction DCM corrects the small misalignment between the two world frames and between successive times when the hinge criterion (described below) is satisfied. The resulting corrected form of Equation (5) becomes,

(9) R(t)ATAS= RATT R(t)TWTC(t)WTWS R(t)WSS RSAS

where $R{\left(t\right)}_{AT/AS}$ is again the needed time-varying direction cosine describing the orientation of the anatomical shank frame relative to the anatomical thigh frame. Note that the ISB recommends [31] first calculating FE, then IE, and finally AA. However, due the mechanical design of the knee analog, the reverse order is required. For the knee analog, the joints are in series order such that the rotation sequence needed to rotate from the shank link to the thigh link require rotations about the AA axis first, the IE axis second, and the FE axis third. Decomposing $R_{AT/AS}$ with that order in mind (analogous to the procedure in [24]) yields the 3D rotation angles across the knee analog.

The above strategy for aligning the two world frames holds for the time intervals when the knee analog in this experiment is predominantly functioning as a hinge joint. To this end, one must develop criteria for determining: (1) when the knee is predominantly functioning as a hinge joint and; (2) a strategy for aligning the two world frames in between those time intervals (i.e., when the knee is no longer predominantly functioning as a hinge joint).

The time intervals when the knee is predominantly functioning as a hinge joint are identified under two conditions: Case (1a) when the knee joint is approximately stationary; and Case (1b) when the knee joint is rotating.

(1a). Functioning as a Hinge—Stationary Case: The knee analog may be considered a hinge joint in the limiting case when it is essentially locked (i.e., straight) and stationary. These time intervals are identified when the segments are stationary and also nominally aligned with gravity (such as the still period in the functional alignment movement sequence). In such instances, the knee functions as a hinge with zero hinge rotation rate. These conditions are considered satisfied in our experiments when,
  (10) $$max\{\left|{\vec{a}}_{T}-\vec{g}\right|,\left|{\vec{a}}_{S}-\vec{g}\right|\}\leq 0.02g$$
  (11) $$mean\left(\cos^{-1}\left(\frac{\vec{a}{\left(t\right)}_{T}\cdot \vec{a}{\left(0\right)}_{T}}{\left|\vec{a}{\left(t\right)}_{T}\right|\left|\vec{a}{\left(0\right)}_{T}\right|}\right),\cos^{-1}\left(\frac{\vec{a}{\left(t\right)}_{S}\cdot \vec{a}{\left(0\right)}_{S}}{\left|\vec{a}{\left(t\right)}_{S}\right|\left|\vec{a}{\left(0\right)}_{S}\right|}\right)\right)\leq 3{}^{\circ}$$
  in which $\vec{a}{\left(0\right)}_{T}$ and $\vec{a}{\left(0\right)}_{S}$ denote the (constant) acceleration measured during the functional alignment step with the segments vertical.
(1b). Functioning as a Hinge—Rotating Case: When the conditions for the stationary case above are not met, the knee joint is considered rotating. During such instances, the knee may still function predominantly as a hinge joint (with non-zero hinge rotation rate) whenever the angular velocities of the shank ${\vec{\omega }}_{S}$ and the thigh ${\vec{\omega }}_{T}$ are predominantly aligned with the hinge axis defined by ${\hat{n}}_{T}$ and ${\hat{n}}_{S}$ on each segment respectively. For our experiment, the knee functions as a hinge with non-zero hinge rotation rate whenever
  (12) $$min\{\left|{\vec{\omega }}_{T}\right|,\left|{\vec{\omega }}_{S}\right|\}\geq 30{}^{\circ}/s$$
  (13) $$mean\left(\frac{{\vec{\omega }}_{T}\cdot {\hat{n}}_{T}}{\left|{\vec{\omega }}_{T}\right|},\frac{{\vec{\omega }}_{S}\cdot {\hat{n}}_{S}}{\left|{\vec{\omega }}_{S}\right|}\right)>0.99$$

The numerical thresholds for the criteria above are stringent, but appropriate for the knee analog we employ in our study. (These thresholds would need to be adjusted for human subject testing as discussed further in the Results and Discussion section.) There are frequent periods of times when neither criteria are met and the knee analog no longer functions purely as a hinge as described next.

(2) Not Functioning as a Hinge: When neither case above holds, the knee is no longer functioning as a hinge and appreciable internal/external rotation and/or abduction/adduction exists. During such time intervals, we assume that $C{\left(t\right)}_{WT/WS}$ varies slowly and continuously and that it can be estimated by linear interpolation between two consecutive “update” times when either of the two cases above hold.

## 3. Results and Discussion

This section contains both qualitative and quantitative comparisons and discussions of the angles estimated using IMU data and the truth data obtained from the CMM. A brief discussion of extending the method for measurements on a human knee concludes the section.

**Figure 2:**
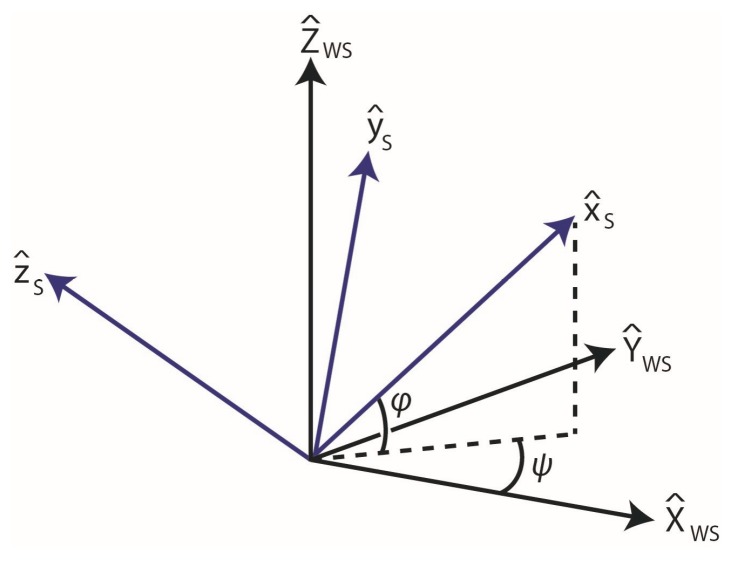
The orientation of the shank IMU frame, $F_{S}$, (blue) relative to the shank world frame, $F_{WS}$, (black). The orientation is defined by the illustrated yaw ψ and vertical φ angular components. The horizontal dotted line is the projection of ${\hat{x}}_{S}$ onto the ${\hat{X}}_{WS}\;$– ${\hat{Y}}_{WS}$ plane and the vertical dotted line is the projection onto the vertical direction.

We begin by noting that the DCMs between the sensor frames and their respective world frames are defined by both horizontal (or yaw) and vertical (or elevation) angular components. Studying these angular components is important for understanding the challenge (and the solution) to constructing the correction DCM $C_{WT/WS}$ introduced above that corrects the differing and drifting IMU world frames. Figure 2 illustrates the orientation of the shank IMU frame $F_{S}$ relative to its world frame $F_{WS}$ in terms of both yaw ψ and vertical φ angular components. In particular, the unit vectors ${\hat{X}}_{WS}$ and ${\hat{Y}}_{WS}$ define the “horizontal” plane for $F_{WS}$, which differ from that of $F_{S}$ by the vertical angle φ. Moreover, the frames also differ by the yaw angle ψ, which is defined as the angle between ${\hat{X}}_{WS}$ and the projection of ${\hat{X}}_{S}$ onto the horizontal plane for $F_{WS}$. One way to visualize the drift error is to examine how either angle varies with time over a trial during which the knee is periodically returned to its initial orientation.

**Figure 3:**
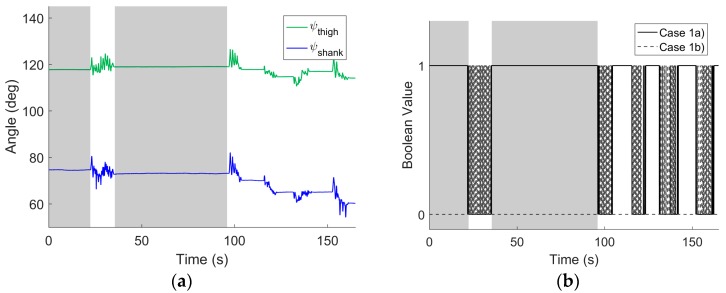
Example results from pure flexion/extension trial. (**a**) Yaw angles for shank- and thigh-mounted IMU versus time encompassing two functional alignment movements (first shaded and unshaded regions, respectively), a rest period (second shaded region), and then four trials of five repetitive knee flexion/extension movements between which the knee is returned to the original position. (**b**) Boolean (0 or 1) values for criteria defining Case 1a (stationary) and Case 1b (rotating) for which the knee analog acts as a hinge; refer to Section 2.3. The solid black line is for Case 1a and the dashed grey line is for Case 1b. The shaded and unshaded areas denote the same regions in (**a**).

For example, Figure 3a illustrates how the yaw angles computed for both IMUs (relative to their respective world frames) vary with time for the simplest testing session when the knee undergoes pure flexion/extension. The portion of the testing session shown encompasses the two functional alignment movements (first shaded interval corresponds to the still period and the first unshaded interval corresponds to FE rotations), followed by another still period (second shaded interval), then a longer (second unshaded) interval with four trials of five repetitive knee flexion/extension movements (with 46 more trials thereafter that are not shown). The knee is approximately returned to its original position at the end of each knee flexion/extension trial. Note that this approximately 3-min sample is taken from a testing session lasting approximately 15 min, and substantial drift arises over this long period of time. Figure 3b illustrates the Boolean values for the criteria for the two cases (Case 1a and Case 1b) when the knee acts as a hinge; a value of 1 corresponds to the criteria being satisfied, and a value of 0 corresponds to the criteria not being satisfied. As expected, the knee analog functions as a hinge for the stationary case (Case 1a) during the rest periods. Similarly, the knee analog functions as a hinge during the subsequent rotations that induce pure flexion/extension for this trial (Case 1b). (There are also short intervals when Case 1b is not satisfied as the angular velocity magnitudes fall below the selected threshold values.)

The orientation drift error manifests in the slowly changing (low frequency) components of $\psi_{thigh}$ and $\psi_{shank}$ despite the fact that the knee returns to its nominal orientation between successive flexion/extension movements. In particular, the net change in $\psi_{thigh}$ and $\psi_{shank}$ over the entire 15-min period are 65° and 101°, respectively. This corresponds to drift rates for the thigh and shank sensors of −0.07°/s and −0.11°/s, respectively, which are consistent with previously measured drift rates for MEMS inertial sensors; see, for example, [34]. The two world frames are misaligned at the start of the trial and continue to drift apart throughout the trial and it should also be noted the drift is not exclusively about the vertical$\;(\hat{z}$) axis. The misalignment of the world frames, and its associated drift, is compounded by sources of ferromagnetic interference in the laboratory environment. Despite the observably large misalignment of the world frames, the method above correctly identifies the correction DCM needed to accurately estimate 3D rotations across the knee analog.

**Figure 4:**
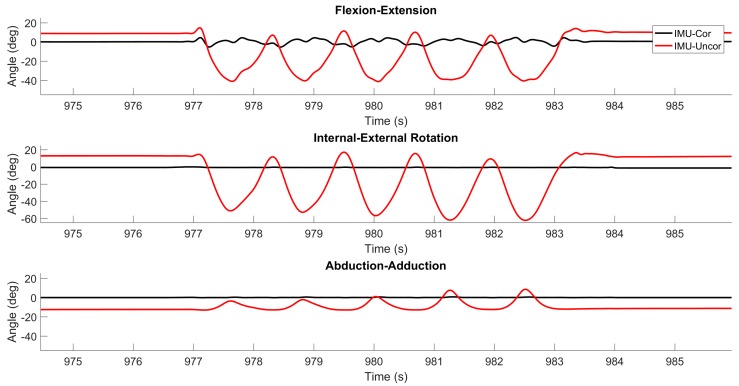
Example results from pure flexion/extension trial. The difference between CMM truth data and IMU-derived estimates with the correction (black) and without the correction (red) of flexion/extension, internal/external rotation, and abduction/adduction are plotted for a representative time period.

Figure 4 illustrates the differences between the true (CMM) and estimated (IMU) flexion/extension, internal/external rotation and abduction/adduction angles for one trial of five repetitive flexion/extension movements previously considered in Figure 3. These estimates closely track the truth values reported by the optical encoders; refer also to quantitative comparison that follows. Also shown are the differences between the true and (uncorrected) estimates that result from using Equation (5) instead of (9), or equivalently, assuming that $C_{WT/WS}=I$. Clearly, ignoring this correction leads to large errors and for all three rotation angles. Note that throughout this trial, the knee analog functions largely as a hinge, meaning that either Case 1a or Case 1b is almost always satisfied (and the linear interpolation associated with Case 2 is not required)—refer to results illustrated in Figure 3b. However, this is not the case in the following trials that induce substantial internal/external rotation and/or abduction/adduction.

**Figure 5:**
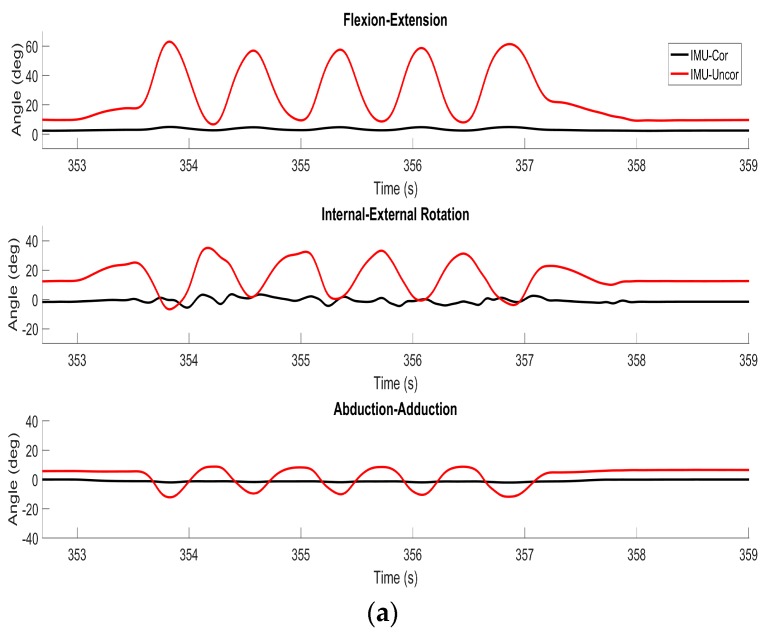

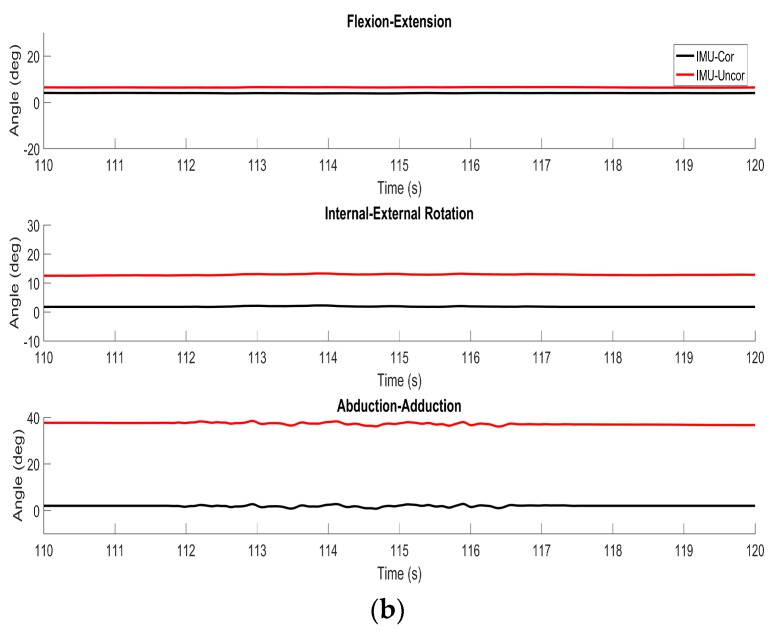
Example results from (**a**) pure internal/external rotation trial, and (**b**) pure abduction/adduction trial. The differences between the CMM truth data and IMU-derived estimates with the correction (black) and without the correction (red) of flexion/extension, internal/external rotation and abduction/adduction are plotted for a representative time period.

Figure 5 illustrates example results for two trials that consider (a) pure internal/external rotation and (b) pure abduction/adduction. As with the prior case, the differences between the true and estimated flexion/extension, internal/external rotation, and abduction/adduction angles remain small, meaning the estimates closely track the truth values reported by the optical encoders. For instance, observe the very slight “off-axis” errors along rotation axes that are otherwise physically constrained on the knee analog. These small off-axis rotations likely derive from very minor errors in the estimated orientations of one or both of the anatomical frames, $F_{AT}$ and $F_{AS}$. Nonetheless, the agreement between the IMU estimates and the truth data remains excellent. By contrast, large errors again arise in general when one ignores the correction. The agreement with the correction for these limiting cases of 1D rotation remains undiminished when the knee analog is unconstrained and undergoes fully three-dimensional rotations as shown next.

**Figure 6:**
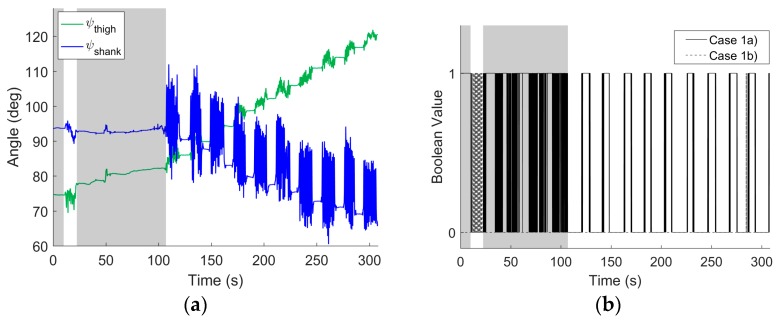

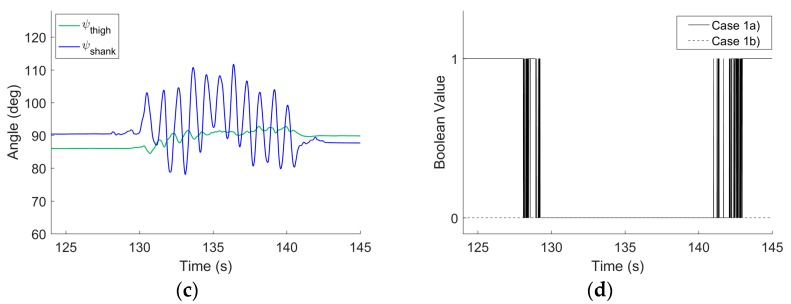
Example results from combined 3D rotation trial. (**a**) Yaw angles for shank- and thigh-mounted IMU versus time encompassing two functional alignment movements (first shaded and unshaded regions, respectively), a nominal rest period during which the constraints are removed (second shaded region), and then ten repetitive 3D movements between which the knee is approximately returned to the original position. (**b**) Boolean (0 or 1) values for criteria defining Case 1a (stationary) and Case 1b (rotating) for which the knee analog acts as a hinge; refer to Section 2.3. The solid black line is for Case 1a and the dashed grey line is for Case 1b. The shaded and unshaded areas denote the same regions in (**a**). In addition, (**c**,**d**) illustrate sample results from (**a**,**b**), respectively, on a fine (second-level) time scale.

We first present a sample time record of the yaw angles for each IMU in Figure 6a when the knee undergoes combined 3D rotations. Following Figure 3, Figure 6 includes a portion of a testing session that encompasses the two functional alignment movements (first shaded and unshaded intervals, respectively), followed by a nominally still period during which the constraints are removed (second shaded interval), then a longer interval with six repetitive 3D movements (with four more thereafter that are not shown). The knee analog is approximately returned to its original position at the end of each knee movement. Note that this is taken from a testing session lasting approximately 6 min, and substantial drift arises over this period of time. Compared to the results of Figure 3, the results of this longer time sample in Figure 6a exhibit even greater drift error as again manifested in the slowly changing (low frequency) components of $\psi_{thigh}$ and $\psi_{shank}$. The net changes in $\psi_{thigh}$ and $\psi_{shank}$ over the entire 6-minute period is 46° and 28°, respectively, which correspond to drift rates of 0.14°/s and −0.08°/s. Despite the very apparent drift, the method produces excellent estimates of the 3D rotations. Figure 6b documents the Boolean values for the criteria for the two cases (Case 1a and Case 1b) when the knee acts as a hinge; a value of 1 corresponding to the criteria being satisfied and a value of 0 corresponding to the criteria not being satisfied. As before, the knee analog functions as a hinge for the stationary case (Case 1a) during rest periods and functions as a hinge during the flexion/extension functional alignment movement (Case 1b). Note that during the second shaded area, the physical constraints are being removed from the CMM in preparation for the combination rotation trials to follow.

**Figure 7:**
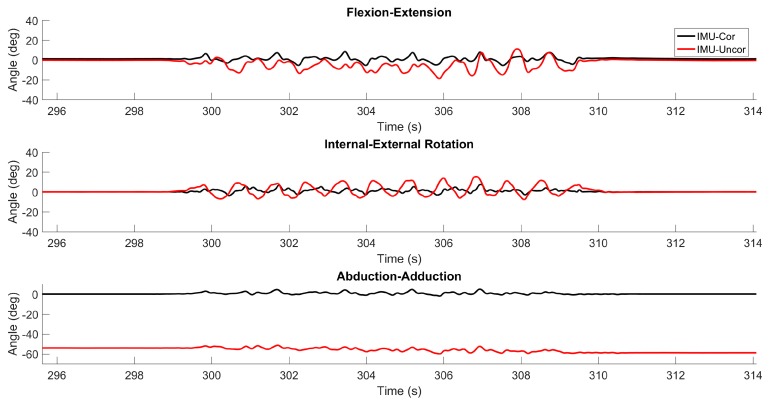
Example results from combined 3D rotation trial. The differences between the CMM truth data and the IMU-derived estimates with the correction (black) and without the correction (red) for flexion/extension, internal/external rotation, and abduction/adduction are plotted for a representative time period.

Figure 7 illustrates the estimated flexion/extension, internal/external rotation and abduction/adduction angles for a representative time period from Figure 6 during which all three angles were being simultaneously exercised. Consistent with the above results, the estimates of all three angles closely track the truth values reported by the optical encoders when the correction is employed. When the correction is not employed, the estimates can be rather poor and particularly so for abduction/adduction in this example.

### 3.1. Quantitative Comparisons

**Figure 8:**
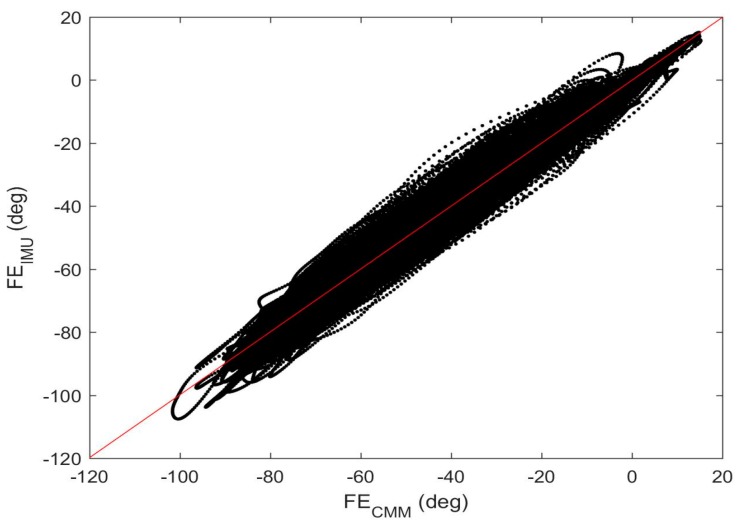
Correlation of the results for the combined 3D rotation trials. IMU-estimated flexion/extension plotted against the corresponding truth data from optical encoder. The red line is the linear fit.

The above results illustrate very close qualitative agreement between the IMU-derived estimates of the joint angles and those measured directly using the embedded optical encoders. Next, we provide quantitative comparisons for the entire data set. To start, consider Figure 8 which shows the IMU-estimated flexion/extension angle versus that measured by the optical encoder for the duration of all five testing sessions (cumulatively about 30 min) of combined 3D rotations. The best fit line to this data yields a slope of 1.01, a y-intercept of 0.07 (°), and a correlation coefficient of *r* = 0.99. Thus, the estimates exhibit extremely high correlation with the truth data (and just slightly over predicting flexion/extension relative to the truth data). In addition, the root-mean square (RMS) error between the estimates and the truth data is 3.46° or 2.96% relative to the 117° range of motion. These results, and the analogous quantitative comparisons for all of the experiments, are summarized in Table 1 and Table 2 below.

Table 1 reports the quantitative comparisons of IMU-estimated angles to those measured by the optical encoders for the three limiting cases of pure rotation about the FE, IE and AA axes. Reported are the range of motion (ROM) about each axis, the RMS error between the estimated and measured angles, and the correlation coefficient, slope and y-intercept of the associated linear fit. Analogous results are reported in Table 2 for the combined 3D rotation movements. Regardless of the trial (pure rotation about any one axis or combined rotation about all three axes), the IMU-derived angle estimates remain within 4° of those measured by the optical encoders, have correlation coefficients exceeding 0.94 and slopes between 0.99 and 1.02. In short, the method yields estimates that replicate the truth data with high confidence. The ranges of motion for each angle are motivated by the behavior of the human knee for which flexion/extension typically has the greatest range of motion, followed by internal/external rotation, and abduction/adduction. However, the knee analog is exercised over far greater ranges of motion than observable on a healthy human knee for the purpose of this thorough validation study.

Although the results presented above demonstrate excellent agreement with the truth data, there are possible remaining error sources in addition to those associated with the correction DCM $C_{WT/WS}$. Referring to Equation (9), errors may arise from estimates of $R_{T/WT}$ and $R_{WS/S}$ that are provided by the chosen Kalman filtering method. As illustrated in Figure 3a and Figure 6a, substantial drift arises about the vertical axis despite the filtering method (and the use of magnetometer data). Errors may also arise from estimates of $R_{AT/T}\;$and $R_{S/AS}$ which, as already noted above, are likely responsible for the very slight off-axis rotations arising in Figure 5.

### 3.2. Extension to Measurements on the Human Knee

The above method yields highly accurate results when benchmarked on a coordinate measurement machine (CMM). It therefore it has considerable potential for accurately estimating the 3D rotations across the human knee. Doing so will also require further research to address additional considerations. First, unlike the CMM used herein, the knee joint has some laxity which may also influence the estimated 3D joint angles. Second, the stringent limits used in Equations (10)–(13), while fully appropriate for the CMM, must necessarily be relaxed for the human knee. Careful validation tests using human subjects will guide the selection of these new limits. Third, while the IMUs were rigidly fastened to the links of the CMM, the IMUs attached to the human shank and thigh may move slightly relative to the underlying skeleton due to soft tissue movements, a problem common to all motion capture methods. The accuracy of the estimated 3D rotations also depends on accurately establishing the orientation of the shank and thigh anatomical frames relative to their respective IMU frames as input to Equation (9). This motivates the need to study a variety of functional alignment movements to achieve this intermediate result.

## 4. Summary and Conclusions

This study contributes a new method to estimate the three-dimensional rotations across the knee using a pair of shank- and thigh-mounted IMUs. Central to this method is constructing a common world frame of reference for the two IMUs by exploiting a vector constraint equation for the medial-lateral axis of the knee. This constraint arises during the time periods when the knee behaves predominantly as a hinge joint, during which and between times the common world frame can be constructed despite sensor orientation drift errors. Estimates of knee flexion/extension, internal/external rotation, and abduction/adduction follow from an estimate of the DCM between the shank and thigh anatomical frames of reference. The method is carefully tested using a coordinate measuring machine that kinematically replicates the 3D rotations across the human knee joint. The embedded optical encoders yield high precision truth data over four qualitatively distinct knee movements spanning (1) pure flexion/extension, (2) pure internal/external rotation, (3) pure abduction/adduction, and (4) combinations of all three rotations. Over N = 50 trials, the RMS error and linear correlation coefficient between the IMU estimates and the optical encoder measurements is 3.90° and *r* = 0.99 for pure flexion/extension, 1.83° and *r* = 0.99 for pure internal/external rotation, and 0.12° and *r* = 0.99 for pure abduction/adduction. During combined movements, the RMS errors and correlation coefficients remain largely unchanged (3.46° and *r* = 0.99 for flexion/extension, 2.48° and *r* = 0.99 for internal/external, and 1.69° and *r* = 0.94 for abduction/adduction.) In summary, the IMU-derived estimates of 3D knee rotations replicate the truth data with high confidence. Our future work includes extending this methodology to trials on human subjects with direct comparison to the 3D knee rotations measured using optical motion capture.

## Figures and Tables

**Table 1:** Quantitative comparisons for cases of pure rotation about a single axis including range of motion (ROM), RMS error, correlation (*r*), and slope and y-intercept (*b*) of linear fit.

1D Rotation	ROM (°)	RMS Error (°)	*r*	Slope	*b* (°)
Pure Flexion/Extension	161	3.90	0.99	1.01	0.12
Pure Internal/External	72.8	1.83	0.99	1.01	0.04
Pure Abduction/Adduction	17.0	0.12	0.99	0.99	−0.02

**Table 2:** Quantitative comparisons for the combined 3D rotation trial including range of motion (ROM), RMS error, correlation (*r*), slope and y-intercept (*b*) of linear fit.

3D Rotation	ROM (°)	RMS Error (°)	*r*	Slope	*b* (°)
Flexion/Extension	117	3.46	0.99	1.00	0.07
Internal/External	98.4	2.48	0.99	1.02	0.06
Abduction/Adduction	58.3	1.69	0.94	1.02	0.04
